# Allosteric modulation of gonadotropin receptors

**DOI:** 10.3389/fendo.2023.1179079

**Published:** 2023-05-25

**Authors:** Clara Lazzaretti, Manuela Simoni, Livio Casarini, Elia Paradiso

**Affiliations:** ^1^ Unit of Endocrinology, Department of Biomedical, Metabolic and Neural Sciences, Baggiovara Hospital, University of Modena and Reggio Emilia, Modena, Italy; ^2^ Center for Genomic Research, University of Modena and Reggio Emilia, Modena, Italy; ^3^ Department of Medical Specialties, Azienda Ospedaliero-Universitaria di Modena, Baggiovara Hospital, Modena, Italy

**Keywords:** FSHR, LHCGR, allosteric modulators, gonadotropins, biased signalling

## Abstract

Gonadotropins regulate reproductive functions by binding to G protein-coupled receptors (FSHR and LHCGR) expressed in the gonads. They activate multiple, cell-specific signalling pathways, consisting of ligand-dependent intracellular events. Signalling cascades may be modulated by synthetic compounds which bind allosteric sites of FSHR and LHCGR or by membrane receptor interactions. Despite the hormone binding to the orthosteric site, allosteric ligands, and receptor heteromerizations may reshape intracellular signalling pattern. These molecules act as positive, negative, or neutral allosteric modulators, as well as non-competitive or inverse agonist ligands, providing a set of new compounds of a different nature and with unique pharmacological characteristics. Gonadotropin receptor allosteric modulation is gathering increasing interest from the scientific community and may be potentially exploited for clinical purposes. This review summarizes the current knowledge on gonadotropin receptor allosteric modulation and their potential, clinical use.

## Activation and allosteric modulation of gonadotropin receptor signalling

**Figure 1:**
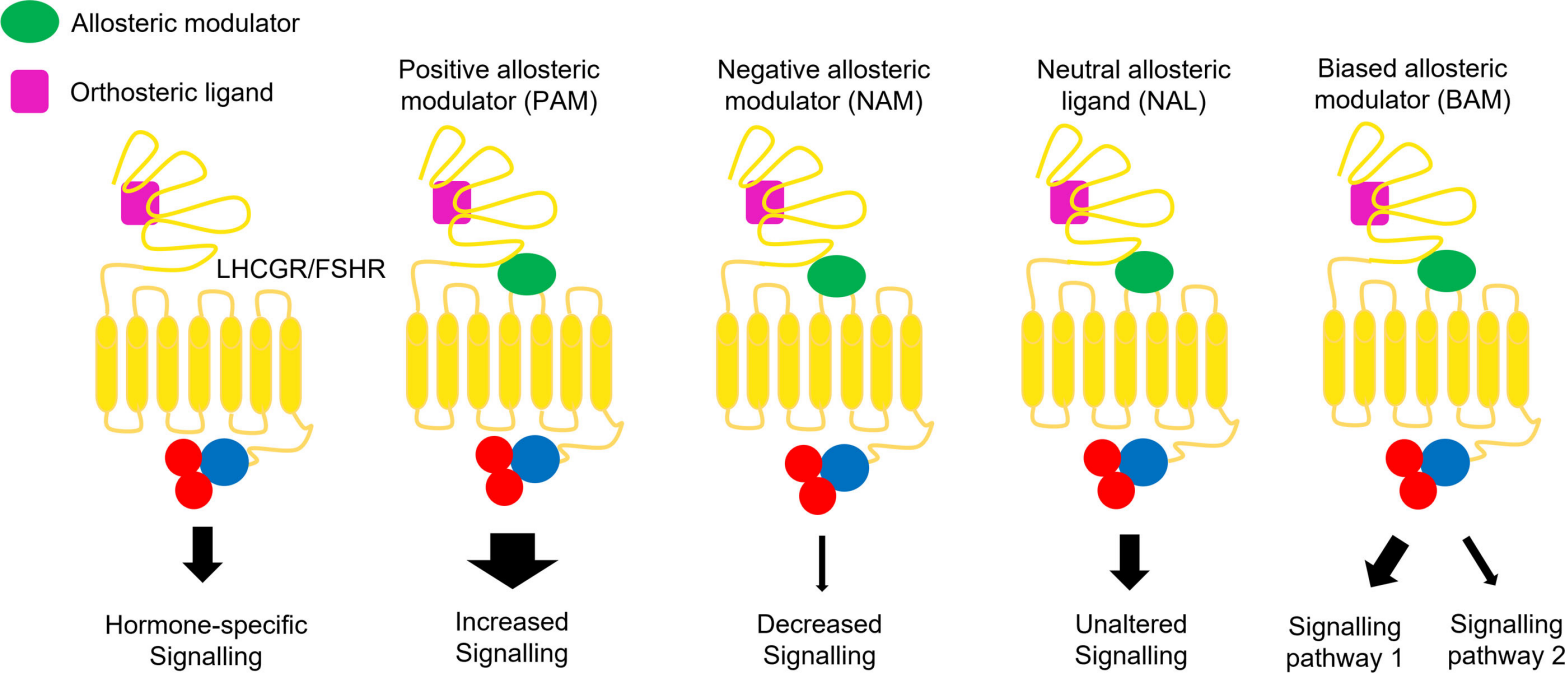
Allosteric modulation of FSHR and LHCGR signalling. Positive allosteric modulators (PAMs) potentiate the agonist-induced signalling, while negative allosteric modulators (NAMs) induce inhibitory effects. Neutral allosteric ligands (NALs) do not affect the ligand activity, while biased allosteric modulators (BAMs) lead to pathway-specific effects.

Luteinizing hormone (LH), follicle-stimulating hormone (FSH) and human chorionic gonadotropin (hCG) are glycoproteins supporting development and reproductive functions (1, 2). LH and FSH are produced by pituitary in a pulsatile fashion and sustain gametogenesis and the conversion of androgens into estrogens. hCG is the pregnancy hormone produced by trophoblast cells and its main action is to mediate progestational effects. These hormones have a receptor-specific β subunit and a common α subunit, shared with the cognate molecule thyroid-stimulating hormone (TSH). LH/hCG receptor (LHCGR) and follicle-stimulating hormone receptor (FSHR) belong to G protein-coupled receptor (GPCR) superfamily. They have a conserved molecular structure characterized by seven helical transmembrane domain (7TMD) linked to a N-terminal extracellular domain (ECD) and a C-terminal intracellular domain. The ECD and the 7TMD are connected with a hinge region, which plays a fundamental role in receptor activation. Also, the 7TMD has three extracellular and three intracellular loops involved in hormone binding and signal transduction, respectively (3, 4). Hormone binding to the orthosteric site induces a conformational change that provokes the activation of multiple intracellular signalling pathways. The structure of LHCGR bound to hCG and the shift from inactive to active state of the receptor were recently resolved by cryo-electron mycroscopy (cryo-EM). Hormone-receptor binding occurs through electrostatic interactions between the hormone and amino acid residues located on the leucine-rich repeats of the ECD (4). hCG binding to LHCGR implies a “push-and-pull” mechanism involving the ECD and the hinge region, impacting the 7TMD spatial conformation (4). It was observed a similar mechanism of FSHR activation, where FSHβ induces the rotation of ECD by pushing it away from the membrane layer, while the hinge region pulls the complex in the active upright conformation by interacting with the α subunit (5–7). Finally, a highly conserved sulfated tyrosine at the hinge loop is fundamental for ligand binding (7). Since it exists in all glycoprotein hormone receptors, it is plausible that the mechanism of receptor activation is highly conserved. In addition to the orthosteric site, GPCRs have a variety of allosteric binding sites for different ligands (**Figure 1**), which may be responsible for conformational changes of the tertiary and quaternary protein structures (8, 9). Due to their structural and sequence similarities, glycoprotein hormone receptors were reported to share important allosteric binding pockets located in the top half part of the TMDs (7). Allosteric modulators may bind their own site and modulate the activity of orthosteric ligands modulating the conformational structure of the active receptor (10, 11). Moreover, they may impact binding affinity or modify signaling patterns mediated by natural ligands (12), or activate the receptor in the absence of orthosteric ligand, resulting in agonistic effect. This is due to a lack of specificity in allosteric binding sites, which are not as highly conserved as the orthosteric site (13). In addition, allosteric binders may modulate or induce receptor trafficking and/or biased signaling (14), and change potency and/or efficacy of the endogenous agonist (15). Finally, these modulators may also lead to saturating effects, limiting, or impairing agonist action (16). Allosterism at the FSHR and LHCGR was also found upon receptor-receptor interaction (6, 17–21) or by binding of specific antibodies (22, 23). Taken together, allosteric modulation of gonadotropin receptors may be induced by different natural and synthetic ligands (**Table 1**) and could naturally occur via interaction with partner membrane receptors. These concepts may be exploited to modulate or bias the signalling network mediated by gonadotropin receptors, for clinical purposes.

**Table 1:** Allosteric modulators of LHCGR and FSHR.

Allosteric name	Mechanism	Testing	Target	Reference
TP4/2	PAM	male Wistar rats	LHCGR	(24)
Org41841	PAM	mouse Leydig cells, immature mice and Cos7 cells	LHCGR/FSHR	(25, 26)
Org43553	PAM	Chinese hamster ovary (CHO) cells, human embryonic kidney 293 (HEK293) cells, rat and human	LHCGR	(27–29)
LUF5771	NAM	CHO cells	LHCGR	(30)
LUF5419	NAM	Rat	LHCGR	(31)
BAY-298 and BAY-899	NAM	CHO cells	LHCGR	(32)
ADX68692 and ADX68693	NAM	Rat, mouse Leydig tumor cell (mLTC-1) and HEK293 cells	FSHR/LHCGR	(33–35)
p,p′DDT	PAM/NAM	CHO cells	FSHR/LHCGR	(36, 37)
thiazolidinones (TZDs)	PAM	CHO cells	FSHR	(38, 39)
Org-21444-0	PAM	Rat, human granulosa cells	FSHR	(40)
MK-8389	PAM	Human	FSHR	(41)
TOP5668 and TOP5300	PAM	Rat, mice, CHO cell, human granulosa cells	FSHR	(42)
ADX61623	NAM	Rat, rat granulosa cell and HEK293 cells	FSHR	(33)
FSHβ (89-97	NAM	Rat, HEK293 cells	FSHR	(43)

Biased signaling is defined as preferential signaling through a specific intracellular interactor (44). Molecules acting as allosteric binders may exert negative or positive modulation of signaling pathways typically triggered by endogenous ligands (8). Considering these issues, positive allosteric modulators (PAMs) are defined as ligand that enhance agonists effects on GPCRs, increasing its efficacy or affinity (45); negative allosteric modulators (NAMs) are non-competitive or inverse agonist ligands attenuating agonist affinity and/or efficacy (46); neutral allosteric ligands (NALs) are those not affecting receptor or orthosteric ligand activity (8). The latter are also called “silent allosteric modulator” and may block the activity of NAMs, PAMs, or allosteric agonists, without exerting any effects by themselves (8, 47, 48). Biased allosteric modulators (BAMs) were identified as molecules activating pathway-specific effects among receptor signaling cascades (49).

This review discusses the most recent updates about allosteric modulation of gonadotropin hormone receptors.

## PAMs on LHCGR

Many small synthetic peptides capable of modulating LHCGR activity have been discovered. They are structurally similar low-molecular weight (LMW) agonists selectively activating intracellular effectors. These molecules which similarly interact with LHCGR, are all potential compounds to be used in clinical practice as alternatives of LH and hCG. Besides a selective activation of the LHCGR-mediated pathways, they often display other useful characteristics such as a greater resistance to peptidase activity than endogenous agonists, which are more vulnerable to degradation and consequently hardly proposed for orally administrable molecules (31). Most of these compounds are thienopyrimidines, which interact with the receptor by binding an allosteric pocket within the TMs of the receptor (4, 24, 28) and activating the intracellular signalling without the involvement of the extracellular domain (28).

The allosteric agonist 5-amino-N-tert-butyl-4-(3-(1-methylpyrazole-4-carboxamido)-phenyl)-2-(methylthio) thieno [2,3-d] pyrimidine-6-carboxylic acid amide (TP4/2) is one of the most recent thienopyrimidines and it was tested in four-month-old, aging (18-month-old) and diabetic male Wistar rats with impaired testicular functions, where the compound was administered both intraperitoneally and subcutaneously for five days. TP4/2 induced the expression of genes coding StAR (*STARD1*) and 17,20-lyase (*CYP17A1*) enzymes, potentially enhancing the steroidogenic signal and without suppressing the expression of the *LHCGR* gene (24). The latter is important to sustain the sensitivity of the testis to endogenous gonadotropins, improving parameters of sperm quality (24). Similar results were obtained with orally administrable LHCGR LMW agonists, which act on the same allosteric site as the other thienopyrimidines. These compounds get close to the transmembrane domain and interact with the transmembrane helices (TMHs) 3, 4, 5, 6 and 7 (4, 50). Because of their similar and sometimes overlapping binding pockets, these PAMs were observed to induced similar effect both *in vivo* and *in vitro*. Org41841 was the first LMW-agonist for a gonadotropin receptor with *in vivo* efficacy after oral administration. This molecule induced testosterone synthesis, in mouse Leydig cells, and ovulation in 40% of immature mice when administered orally. Moreover, it induces an upregulation of FSHR expression (25, 26). However, one of the main issues linked to these allosteric modulators are the lack of specific action on LHCGR due to the high homology among glycoprotein hormone receptors. Org41841 was indeed demonstrated to have a binding capability and agonistic activity towards the thyroid-stimulating hormone receptor (TSHR), which shares with LHCGR the highly conserved pocket where Org41841 interact with the receptors (4, 50). Another LHCGR PAM is Org43553 (19, 51), which belongs to the group of thienopyrimidines and interacts with a LHCGR binding pocket located between TMH 3, 5, 6 and 7 (4, 31) as well as residues of extracellular loop (ECL) 2 and 3 and the receptor hinge region (4) The first evidence of Org43553 modulatory activity through the TMD of LHCGR was demonstrated by two chimeric TSHR and LHCGR with the extracellular domains of the two receptors exchanged. Only the wild type LHCGR and the chimeric receptor with the LHCGR TMD could mediated the intracellular signalling in response to Org43553 treatment, demonstrating both the specific action of the PAM on LHCGR and its interaction site on TMD (28). Particularly relevant for the allosteric modulator action are the residues A589 and I585, whose mutations cause a reduced Org43553 functional activity (4). This compound triggers LHCGR-mediated intracellular cAMP increase in Chinese hamster ovary (CHO) cells, but not LHCGR-mediated phospholipase C (PLC) activation in HEK293 cells expressing human LHCGR. Org43553 strongly inhibited LH-induced inositol phosphate (IP) production in a non-competitive manner, showing biased agonism at the receptor (28). Moreover, Org43553 may be administered orally, and has high oral bioavailability with short half-life (between 30 and 47 h) (52). Experiments in rodents revealed that this PAM may induce ovulation in females and increases serum testosterone in males (27, 29, 53). Most importantly, the clinical use of Org43553 was suggested in women undergoing assisted reproduction, to reduce the potential risk of ovarian hyperstimulation syndrome (OHSS). This is a pathological condition that may occur upon administration of hCG to induce ovulation. Since Org43553 has shorter half-life than hCG, it is plausible that the use of this PAM could be associated with lower OHSS risk, but this issue needs to be clinically confirmed (54).

## NAMs on the LHCGR

Unlike LHCGR agonists, the number of LHCGR antagonists is very limited. LUF5771 was the first LMW NAM acting on LHCGR (30). LUF5771 may displace both hCG and Org43553 from the receptor and reduce the expression of a *CRE* promoter-controlled reporter system (30, 31). Interestingly, these effects were decreased 2- to 10-fold in the presence of another modulator (LUF5419), which can enhance Org43553 binding and efficacy as well (30). Structural receptor modelling studies predicted the existence of two different allosteric binding pockets located in the upper portion of the TMD, that partially overlap, and which may explain the activating or inhibitory effects of the compounds on the receptor. While LUF5771 has a binding site located between TMHs 1, 2, 3, 6, 7 and the ECL2, Org43553 interact with the receptor at the TMHs 3, 5 and 6 level. LUF5419 shares with LUF5771 the same binding pocket, however it does not interact with TMH 1 and 2, showing no inhibitory effect (30, 32). These structural predictions underline that the inhibitory effect may require TMH 2 and 7 by keeping the receptor in the inactive state (30). In conclusion, compounds displaying antagonist activity at the LHCGR exist, but further studies are needed to characterize their full mode of action and, eventually, to develop binders with different effects.

Recently, two molecules with potent LHCGR negative allosteric activity have been identified. They are tetrahydro-1,6-naphthyridine-based compounds namely BAY-298 and BAY-899, suitable for application in animal models and able to reduce LH-dependent sex steroids serum levels. BAY-298 binds to a site different to that of LH and Org43553, since its binding to the receptor does not compete either with the hormone and the allosteric modulator (32), confirming what previously observed with other NAMs (30). In female rats, BAY-298 reduced LH-mediated gene expression, while the compound inhibited production of testosterone in males. Moreover, it led to cycle arrest in diestrus and metestrus, interrupting folliculogenesis in a dose-dependent manner. BAY-899 was tested in female rats as well, demonstrating that it may reduce serum estradiol levels (32). Other compounds have been designed to modulate LHCGR activity, however they resulted in a low specificity for the receptor, showing cross-reactivity with FSHR as well and diverse actions depending on the cell model analyzed. Two benzamide compounds, ADX68692 and ADX68693 with structural similarities to N-[4-(Cyano-dimethyl-methyl)-phenyl]-3,4-dimethoxy-benzamide (ADX61623), act as NAMs on the FSHR, leading to different effects on steroidogenesis and ovulation. In particular, ADX68692 blocked FSHR-promoted cAMP, progesterone and estradiol production, while ADX68693 inhibited cAMP and progesterone production with similar efficacy as ADX68692, but not estradiol production in rat primary granulosa cells *in vitro* (33, 34). Moreover, both compounds antagonized LHCGR signalling in several cell models, such as human embryonic kidney (HEK293), murine Leydig tumor cell lines (mLTC-1) and rat primary Leydig cells (35). Benzamides inhibited the hCG-mediated cAMP production, although ADX68693 was more potent than ADX68692. Interestingly, both molecules had negative allosteric behavior on hCG-induced β-arrestin 2 recruitment and this effect occurs very rapidly. The impact of ADX68692 and ADX68693 was tested in Leydig primary cells and cell lines. Although these benzamides did not modulate hCG-induced progesterone production in mLTC1 cells, different results were obtained in rat primary Leydig cells. In this cell model, hCG-induced progesterone levels increased in the presence of ADX68692, while the opposite effects were observed upon ADX68693 administration. ADX6892 led to partial inhibition of testosterone synthesis, while ADX68693 completely abolished hCG-promoted testosterone production. These results suggest that both compounds lead to biased effects on intracellular signalling pathways mediating steroidogenesis, since both molecules blocked the canonical Gs/cAMP pathway but not testosterone production (35). To date no structural studies have been carried on ADX68692 and ADX68693 interaction with the two gonadotropin receptors, however it might be supposed that they bind a conserved binding pocket common to the receptors, leading to a similar inhibitory effect on the mediated signalling. Conversely other small molecules designed with a NAM activity were observed to act as PAM as well. In particular, dichlorodiphenyltrichloroethane (p,p′DDT) is an endocrine-disrupting chemical that targets FSHR, increasing the cAMP response to FSH in CHO cells (36). p,p′DDT acts as a LHCGR NAM as well, decreasing the hCG-induced cAMP production in CHO cells (37). Moreover, this compound reduced LH- and hCG-induced β-arrestin 2 recruitment and progesterone production, without altering testosterone secretion (37).

The discovery of different NAMs and their combinatorial use may be exploited for contraceptive or therapeutic applications, such as the development of a specific oral contraceptive with lesser side effects than the currently available steroidal-based drugs.

## PAMs on FSHR

Several small molecule ligands have been identified that interact with FSHR, modulating the hormone binding affinity and related signaling network (55). Different classes of chemicals may act as PAMs, allosteric agonists, NALs or NAMs (55, 56). While PAM and NAM activities are subordinate to FSH binding to the receptor, agonists exert their effects even in the absence of the natural ligand (56, 57). Indeed, allosteric modulators barely interact with the N-terminal region of the receptor, while most of these molecules bind pockets allocated in the TMD of the receptor. A recent study using molecular docking analysis identified four different allosteric binding sites which seem to be specific for the enhanced or inhibitory activity of the allosteric modulators (58). In fact, the binding site situated between TMH 6 and 7 is predicted to be targeted just by NAM molecules, while the others can be bounded both by NAMs and PAMs (58). These findings underline that the biological activity of the allosteric modulator may depend on the specific interaction with residues located in different TMHs, such as Asp521 which emerged to be particularly relevant for both NAMs and PAMs function (58).

The first compounds described as modulators of FSHR activity were the thiazolidinones (TZDs). They were found in a library of combinatorial chemical scaffolds screened by FSHR reporter assay (59). This study identified three thiazolidinone compounds, defined as compounds 1, 2 and 3, interacting specifically with FSHR, without inducing cross-reaction with other glycoprotein hormone receptors, and not competing with FSH for the hormone binding site (38). Structural analysis performed using FSHR/TSHR chimeric mutants revealed that all these compounds interact with a region located within the FSHR TMH1 to TMH3 (38). Compounds 2 and 3, thiazolidinone analogues, were produced by introducing structural modifications to compound 1, thereby obtaining a partial agonist and an antagonist, respectively. These compounds might be assumed to contact differently the TMHs conferring a diverse biological activity despite their common structural origin. The agonist activity of compound 1 was demonstrated by *in vitro* experiments to trigger Gαs protein activation, inducing intracellular cAMP increase (39), phosphorylation of AKT and ERK1/2, and estradiol synthesis, favoring the expansion of ovarian cumulus cells and follicular growth (60). Compound 3 leads to Gαi protein activation, inhibiting cAMP and estradiol production (39, 57, 61).

One of the first orally administrated compounds with FSH-like activity was the dihydropyridine Org-214444-0, which interacts with the receptor in an allosteric binding site consisting in the top half of TMH 5, 6, 7, the ECL2 and 3, and the hinge region (7). The Cryo-EM structure of FSHR gave a further confirmation of the molecular similarities and the conserved binding site of the gonadotropin receptors (7). The analysis performed on Org-214444-0 demonstrated that the FSHR binding pocket targeted by this PAM involves the same TMH and ECL which shape one the LHCGR allosteric binding sites (4). Experimental data demonstrated that it is a lipophilic compound acting as a PAM, inducing a cAMP response and estradiol production in human granulosa cells (40). Interestingly, the molecule supported follicular development in rats (57). This PAM is capable of inducing cAMP production and sustaining follicle maturation in rats (40).

The dihydropyrroloisoquinoline MK-8389 was the first oral FSHR agonist tested in humans. It was used in clinical trials, in young women, where it failed to support ovarian follicular growth, yet exhibited side effects, interfering with thyroid hormone release. As previously mentioned, one of the most limiting aspects to overcome in the development of molecules useful in clinic is the structural similarities of the glycoprotein receptors and their allosteric binding site, which may lead to side effects *in vivo*. Indeed, high doses of MK-8389 resulted in a dose-dependent decrease in thyroid stimulating hormone (TSH) followed by dose-dependent increases in free triiodothyronine (FT3) and thyroxine (FT4) (41, 57). Two other oral FSHR allosteric agonists, TOP5668 and TOP5300, were proposed for ovarian stimulation in assisted reproduction, and tested both *in vitro* and *in vivo* (42). In rats and mice, both molecules enhanced estradiol production, which reached higher levels than those achieved upon treatment by recombinant FSH (42). Moreover, follicular development, oocyte number, fertilization rate, and hatched blastocyst rate, were similar between FSH- and TOP5668/TOP5300-treated animals. Among the population that may benefit of TOP5668 and TOP5300 treatments there are PCOS patients: in the same study, it has been demonstrated that granulosa cells, collected from women affected by PCOS, and treated with TOP5300 produce an increased amount of estradiol compared to the cells treated with the recombinant FSH, commonly used in clinic (42). Conversely, this difference in estradiol production induced by TOP5300 and recombinant FSH was not observed in human granulosa lutein cells isolated from patients with normal ovarian reserve (42). Authors also indicated no issues for safety and toxicity profiles, suggesting these chemicals as potential candidates for clinical trials (42). Other compounds demonstrated to interact with and modulate FSHR activity are benzamide and thiazolidinone derivates, which induce biased signalling. They are linked to different kinetic profiles than FSH, measured as G protein activation, β-arrestin recruitment, and trafficking of the receptor (61) and display properties useful in pharmacology (61, 62). Indeed, to date having molecules able to bias signaling at the FSHR represent important pharmacological tools to understand the specific FSHR signaling pathway contributing to different pathologies both in women and men, such as PCOS and male idiopathic infertility.

New findings on FSHR structure and chemical interactions at the allosteric sites helped to identify specific regions that are involved in the PAM and NAM activity, which can be ascribable to allosteric functions. Recent studies proposed the use of antibodies as modulators of the receptor activity. Antibodies against FSH and/or FSHR have been isolated and developed in order to provoke conformational changes impacting their functions (22). A monoclonal antibody targeting an amino acid sequence located at the FSHβ interaction site with FSHR is reported to block hormone binding. This molecule was tested *in vivo*, suggesting it may prevent bone loss in ovariectomized mice (63). Other antibodies were developed against portions of the FSHR. Three different immunoglobulins targeting decapeptides on the N-termini of FSHR were described about two decades ago. Two of these molecules behaved as antagonists and inhibited cAMP production, while the third did not impede ligand binding. Rather, they acted as agonists, promoting cAMP response (64). Other studies described antibodies acting against the hinge region of different glycoprotein hormone receptors, displaying agonistic activity (14, 65). These antibodies specific for the hinge region stimulated the receptor by bypassing the hormone, but did not influence hormone binding, suggesting that the C-terminal portion of the ECD is not directly involved in hormone binding (66). The recent Cryo-EM structure of the glycoprotein hormone receptors have confirmed the crucial role of the hinge region in the activation of the receptor and the signal transduction (4, 7), suggesting that antibodies targeting this region can modulate the intracellular signalling in a positive or negative manner, without competing with the hormone (67–69). These compounds targeting the hinge region may represent an alternative approach to allosteric chemicals for the treatment of infertility and controlled ovarian stimulation for *in vitro* fertilization. Moreover, the application of nanobodies selected from synthetic libraries were proposed to modulate FSHR activity. This approach may lead to the selection of a large number of candidates not competing with FSH for receptor binding and acting as allosteric modulators (69).

## NAMS on FSHR

Many compounds that reduce or inhibit the receptor activity have been described, which behave as NAMs (14, 57, 65). These molecules are of potential clinical interest because they could serve as contraceptives with a specific and exclusive action on the ovary, avoiding side effects classically associated with hormonal contraception (35). Among NAMs, tetrahydroquinolines are reported to inhibit FSH-induced cAMP production *in vitro*, although they failed to elicit the same activity *in vivo* (70). Other allosteric FSHR antagonists, are the two previously mentioned benzamide compounds ADX61623 and ADX49626 (33, 70). While the first one blocked the FSH-induced cAMP accumulation and progesterone production, but not the estradiol secretion in granulosa cells, ADX49626 behaved as a pure antagonist inhibiting the whole FSH-induced signalling network (33). ADX61623 was recently used to perform studies on FSHR binding to its allosteric modulators. This well-known NAM blocks the receptor in a much more stable position compared to the unbound receptor. It binds the allosteric site which includes residues on TMH 4, TMH 5, TMH 6, EL2 and EL3, among which F503 of TMH4, N521 of EL2 and K598 and V599 of EL3 seems to be particularly relevant for ADX61623 and other NAMs to inhibits FSHR activity (58). Interestingly, despite their negative effect on the FSHR-mediated signalling, they both are reported to increase the number of hormone molecules bound to FSHR, as a concept suggestive of FSHR trimeric formations occurring in the presence of these antagonists, which might influence the homomeric forms of the receptor and consequently the receptor mediated-signalling (71). The two structurally similar compounds, ADX68692 and ADX68693, were later described as both LHCGR and FSHR NAMs (34, 35). In rat granulosa cells, ADX68692 inhibited FSH-dependent cAMP, progesterone, and estradiol production. Conversely, ADX68693 decreased cAMP and progesterone responses but displayed a synergistic effect with FSH on estradiol synthesis. However, only ADX68692 demonstrated efficacy *in vivo*, blocking the follicular growth in rat granulosa primary cells (34). However, none of those molecules were tested on humans. These compounds can act on both gonadotropin receptors, underling one of the main issues of LMW molecules, or rather, their unspecific action due to structural similarities of the allosteric sites of the receptors.

A short peptide was then developed from homology and structural studies on the hFSH-FSHR (ECD) complex, which identified a sequence of the FSHβ seat-belt loop with antagonistic properties on FSH activity (43). This molecule is a peptide named “FSHβ (89-97)” and prevents the [^125^I]-FSH binding to the receptor, and intracellular cAMP accumulation. Moreover, in immature rats, it reduced the ovarian weight gain mediated by FSH, blocked follicle transition from pre-antral to antral stages, and inhibited estradiol production (43). Concerns on the clinical use of FSHβ (89-97) raised from the side-effect observed in rats, where the peptide generated a PCOS-like state that needs further investigation (72).

Eventually, modulation of FSHR function may be mediated by endocrine disruptors (EDs). These are defined as natural or synthetic molecules that interfere with the regulation of the endocrine system. Some of them may act by binding gonadotropin receptors, impacting intracellular signaling pathways. An example is provided by the 1-chloro-4-[2,2,2-trichloro-1-(4-chlorophenyl)ethyl]benzene (p,p’-DDT), which acts as a FSHR PAM. It specifically interacts with a unique amino acid sequence in receptor transmembrane helices 3 and 7. This interaction leads to increased FSHR sensitivity to hCG, which is not the physiological ligand of this receptor (36).

## Allosterism due to receptor heteromerization

In previous sections it was underlined the relevance of the single interactions with specific residues located on the diverse transmembrane domains of the gonadotropin receptors, showing their crucial role in the modulation of the intracellular signal mediated by the receptor itself. Thus, it might be assumed that molecular interactions at the TMHs level, which can occur among receptors at the cell membrane can induce a biased signaling and an allosteric modulation of the receptor. Several GPCRs are known to form complexes of receptors, consisting of homomeric and heteromeric assemblies, which may functionally cooperate on the cell surface (73–76). The formation of homo/hetero-oligomers may lead to allosteric modulation of GPCR functions and bias the intracellular signaling network (18, 23, 76, 77). Despite the *in vivo* existence of these receptors structures is still unknown, relevant *in vitro* effects on the activation of selective intracellular pathways have been reported, suggesting their physiological relevance.

LHCGR-FSHR interactions occur through contacts between amino acid residues in the extracellular domain and multiple transmembrane helices (23, 78, 79). At the interface between heteromeric complexes, electrostatic interactions generate more stable conformations than in homomers. An example of these assemblies is provided by adenosine A2A receptor (A2AR) and dopamine D2 receptor (D2R) heteromerization (80, 81). These interactions may impact hormone binding to the receptor, since each functional unit of the assembly may display a different affinity for its ligand (18, 74).

In the ‘80s, it was reported via electron microscopy images that LHCGR may form clusters in the surface of rat theca and granulosa cells, playing a role in cellular responsiveness to the hormone (82, 83). In later years, the improvement of the existing detection methods and development of new techniques allowed investigators to achieve a deeper knowledge of FSHR-FSHR and FSHR-LHCGR interactions (83). Recent data indicated that specific amino acids residues of these molecules act as contact sites for the partner receptor and they could be promising and innovative targets for new therapeutic approaches for infertility treatments (65, 83, 84). It is worthy of note that interface interaction sites of gonadotropin receptors are mainly located on TMH 5, 6, and 7, which are part of the identified FSHR and LHCGR allosteric sites. Indeed, the previous listed allosteric modulators bind the gonadotropin receptors at the TMD level through molecular interactions which occur in receptor-receptors complexes as well (4, 7, 58, 65, 83).

The impact of LHCGR and FSHR homomers on signal transduction was evaluated in the transfected human embryonic kidney (HEK293) cell line, after coexpression of a ligand binding defective mutant FSHR and a signalling defective mutant FSHR. Upon treatment, the activation either of cAMP or inositol trisphosphate (IP3) occurred, but not both of them, suggesting the mutant receptor pairs stabilize different FSHR conformations and consequently a different interaction with G proteins (20, 83). Interestingly, it seems that some allosteric modulators can affect the assembles formation of FSHR trimers and increase the FSH-binding to the receptors complexes, suggesting that they can exert their modulation in a more complex way then simply binding to the receptor (71). A similar signaling analysis was performed following coexpression of ligand binding and signalling defective LHCGRs. In that system, LH failed to activate the Gq-mediated pathway associated with the LHCGR functional complementation mutants, while there were no differences in the cAMP responses mediated by the wild-type LHCGR and functional mutant receptors (79, 85). Evidence of homodimerization *in vivo* was obtained in genetically modified mice, where the wild-type LH receptor (Lhr) expression was replaced by Lhr defective mutants that could mediate the LH signalling, preventing hypogonadal phenotype, and sustaining normal testosterone levels (86).

*In vitro* studies demonstrated that LHCGR and FSHR form heteromeric structures in the cell surface (18, 65, 87). As a result of this interaction, authors found the attenuation of gonadotropin-induced cAMP increase, likely due to low Gαs protein activation (18). Conversely, co-expression of both receptors induced a higher Gq protein activation. Indeed, the unbound FSHR acts as an allosteric modulator by altering the profile of Gαq/11-dependent Ca^2+^ signalling induced by LH and hCG through LHCGR (23). Different behavior of FSHR-LHCGR heteromers might occur as a function of the expression level for each receptor at the cell surface. The stoichiometric proportion between FSHR and LHCGR molecules may impact the signalling network, triggering the preferential activation of specific intracellular pathways (23, 83, 88). FSHR–LHCGR heterodimers are still hardly detectable *in vivo* due to lack of specific detection methods in living tissues (76).

A recent study demonstrated that FSHR and LHCGR have another interacting partner in the ovary, which is a G protein-coupled estrogen receptor (65, 89). GPER is a class A GPCR expressed in granulosa cells during the follicular stage of the menstrual cycle. It forms heteromeric structures with FSHR, potentially playing a central role in the regulation of ovarian function. The physical interaction between FSHR and GPER leads to displacement of Gαs coupling to the gonadotropin receptor, preventing FSH-dependent cAMP intracellular increase (89). This effect is mediated by an inhibitory complex, consisting of the MAGUK, AKAP5 and PKA protein aggregate, which is bound to the GPER C-terminus. While on the one hand, this complex blocks the Gαs protein signaling, on the other hand, it is unable to inhibit the βγ subunit. As a result, treatment of FSHR/GPER-coexpressing cells by FSH results in the βγ-dependent AKT phosphorylation and activation of survival signals. This molecular mechanism re-shapes steroidogenic and apoptotic signals linked to FSHR/cAMP into proliferative and anti-apoptotic events (89). Interestingly, these data are suggestive of a possible role of heteromers in hormone-responsive cancers co-expressing FSHR and GPER, where the activation of proliferative events may contribute enhanced cell growth (65).

*In vitro* evidence shows an important and selective modulation of the receptors-mediated intracellular pathways, demonstrating the allosteric effect of molecular interactions that occur in heteromeric structures. These findings indicate the potential physiological relevance of those receptor complexes, which may be considered as potential targets of specific drugs.

## Therapeutic potential of gonadotropin receptor allosteric modulators

The development of orally administrable gonadotropin analogues and LMW-compounds active on gonadotropin receptors would be useful for novel therapeutical approaches. In the context of human fertility, these drugs have half-lives longer than hormones, thereby avoiding multiple injections and potentially reducing OHSS risk. This syndrome may occur during hCG treatment. hCG has a relatively long half-life (several hours) that may lead to overstimulation of the ovaries, which could have an exaggerated reaction by swelling and leaking fluid intraperitoneally (90). This effect might be mitigated by the use of long-acting allosteric modulators, which may protect from OHSS risk during the treatment with hCG (91). Among the known LHCGR agonists, the thienopyrimidine Org43553 has a shorter half-life than hCG (3.4 h) and may induce ovulation, reducing the incidence of OHSS (29, 54). In addition, Org43553 has been tested in humans. In the presence of gonadotropins, the compound favored ovulation and follicle maturation, both in pituitary-suppressed and normal healthy women, without producing side effects (19).

LMW modulators could be used in the context of other pathological conditions, such as PCOS, which is characterized by impaired menstrual cycles, formation of multiple cysts in the ovary and excessive production of androgens (92). The availability of allosteric compounds active at the FSHR and LHCGR could modulate gonadotropins’ activity, potentially relieving PCOS symptoms. PCOS patients has been suggested as the potential target population for the use of the TOP5300 in assisted reproduction (42). Authors demonstrated *in vitro* that TOP5300 is more effective than the recombinant FSH in inducing an estradiol production, only in the human granulosa cells isolated from PCOS women, suggesting a specific effect on these patients (42). Similar compounds could be involved in the androgen conversion into estrogens, ameliorating the hyperandrogenic condition which affect these patients. Moreover, allosteric modulators could offer a novel contraception method. The development of orally active FSHR antagonists could be used as substitutes for classical oral steroid-based contraceptives, which may lead to side effects, such as venous thromboembolism, headache, etc. (93). In contrast, allosteric compounds may be used to improve fertility treatments, which currently require daily injection of recombinant or purified gonadotropins. Long-lasting, orally administrable drugs could be useful to reduce the number of drug administrations, improving the comfort of patients undergoing assisted reproduction (53, 65).

Despite several attempts to introduce gonadotropin receptors allosteric modulators in clinical practice after clinical trials, up to now, they are still not commercially available because of their low specificity. Structural and mechanistic studies on gonadotropins and their receptors may contribute to improve the development of small molecule drugs targeting GPCRs.

## Conclusions

Gonadotropin receptors are capable of biased signalling, depending on structural features of the ligand and interactions with partner molecules. These properties have been increasingly studied and used to develop compounds that selectively activate specific intracellular signaling pathways. Gonadotropin receptor allosteric modulation has been demonstrated to occur though heteromeric structures as well, which can deviate, amplify or suppress the hormone-dependent signaling. The different effects of those molecular interactions could find clinical applications to improve treatments of male and female infertility as well as gonadic disorders (94). For this purpose, new oral contraceptives with no side effects may provide useful tools to develop comfortable personalized treatments.

## Author contributions

CL and EP conceived and wrote the manuscript, and reviewed the scientific literature. MS and LC drafted the manuscript. All authors contributed to the article and approved the submitted version.

## References

[1] Simoni M Gromoll J Nieschlag E. The follicle-stimulating hormone receptor: biochemistry, molecular biology, physiology, and pathophysiology. Endocrine Rev (1997) 18(6):739–73. doi: 10.1210/EDRV.18.6.0320 9408742

[2] Ascoli M Fanelli F Segaloff DL. The lutropin/choriogonadotropin receptor, a 2002 perspective. Endocrine Rev (2002) 23(2):141–74. doi: 10.1210/EDRV.23.2.0462 11943741

[3] Kristiansen K. Molecular mechanisms of ligand binding, signaling, and regulation within the superfamily of G-protein-coupled receptors: molecular modeling and mutagenesis approaches to receptor structure and function. Pharmacol Ther (2004) 103(1):21–80. doi: 10.1016/j.pharmthera.2004.05.002 15251227

[4] Duan J Xu P Cheng X Mao C Croll T He X. Structures of full-length glycoprotein hormone receptor signalling complexes. Nature (2021) 598(7882):688–92. doi: 10.1038/s41586-021-03924-2 34552239

[5] Fan QR Hendrickson WA. Structure of human follicle-stimulating hormone in complex with its receptor. Nature (2005) 433(7023):269–77. doi: 10.1038/NATURE03206 PMC5514322 15662415

[6] Jiang X Liu H Chen X Chen PH Fischer D Sriraman V. Structure of follicle-stimulating hormone in complex with the entire ectodomain of its receptor. Proc Natl Acad Sci United States America (2012) 109(31):12491–6. doi: 10.1073/PNAS.1206643109 PMC3411987 22802634

[7] Duan J Xu P Zhang H Luan X Yang J He X. Mechanism of hormone and allosteric agonist mediated activation of follicle stimulating hormone receptor. Nat Commun (2023) 14(1):519. doi: 10.1038/S41467-023-36170-3 36720854 PMC9889800

[8] Christopoulos A. Advances in G protein-coupled receptor allostery: from function to structure. Mol Pharmacol (2014) 86(5):463–78. doi: 10.1124/MOL.114.094342 25061106

[9] Draper-Joyce C Furness SGB. Conformational transitions and the activation of heterotrimeric G proteins by G protein-coupled receptors. ACS Pharmacol Trans Sci (2019) 2(4):285–90. doi: 10.1021/acsptsci.9b00054 PMC7088962 32259062

[10] Katritch V Cherezov V Stevens RC. Structure-function of the G protein-coupled receptor superfamily. Annu Rev Pharmacol Toxicol (2013) 53:531–56. doi: 10.1146/ANNUREV-PHARMTOX-032112-135923 PMC3540149 23140243

[11] Bartuzi D Kaczor AA Matosiuk D. Signaling within allosteric machines: signal transmission pathways inside G protein-coupled receptors. Molecules (Basel Switzerland) (2017) 22(7):1188. doi: 10.3390/MOLECULES22071188 28714871 PMC6152049

[12] Lane JR Abdul-Ridha A Canals M. Regulation of G protein-coupled receptors by allosteric ligands. ACS Chem Neurosci (2013) 4(4):527–34. doi: 10.1021/cn400005t PMC3629737 23398684

[13] Gentry PR Sexton PM Christopoulos A. Novel allosteric modulators of G protein-coupled receptors. J Biol Chem (2015) 290(32):19478–88. doi: 10.1074/JBC.R115.662759 PMC4528113 26100627

[14] Landomiel F De Pascali F Raynaud P Jean-Alphonse F Yvinec R Pellissier LP. Biased signaling and allosteric modulation at the FSHR. Front Endocrinol (2019) 10:148. doi: 10.3389/fendo.2019.00148 PMC6425863 30930853

[15] Kenakin TP. Biased signalling and allosteric machines: new vistas and challenges for drug discovery. Br J Pharmacol (2012) 165(6):1659–69. doi: 10.1111/j.1476-5381.2011.01749.x PMC3372820 22023017

[16] Keov P Sexton PM Christopoulos A. Allosteric modulation of G protein-coupled receptors: a pharmacological perspective. Neuropharmacology (2011) 60(1):24–35. doi: 10.1016/j.neuropharm.2010.07.010 20637785

[17] Casarini L Riccetti L Paradiso E Benevelli R Lazzaretti C Sperduti S, Two human menopausal gonadotrophin (hMG) preparations display different early signaling *in vitro*. Mol Hum Reprod (2020) 26 (12):894–905. doi: 10.1093/molehr/gaaa070 33084890

[18] Feng X Zhang M Guan R Segaloff DL. Heterodimerization between the lutropin and follitropin receptors is associated with an attenuation of hormone-dependent signaling. Endocrinology (2013) 154(10):3925–30. doi: 10.1210/EN.2013-1407 PMC3776865 23825122

[19] Gerrits M Mannaerts B Kramer H Addo S Hanssen R. First evidence of ovulation induced by oral LH agonists in healthy female volunteers of reproductive age. J Clin Endocrinol Metab (2013) 98(4):1558–66. doi: 10.1210/JC.2012-3404 23515453

[20] Ji I Lee C Jeoung M Koo Y Sievert GA Ji TH. Trans-activation of mutant follicle-stimulating hormone receptors selectively generates only one of two hormone signals. Mol Endocrinol (Baltimore Md.) (2004) 18(4):968–78. doi: 10.1210/ME.2003-0443 14726491

[21] Urizar E Montanelli L Loy T Bonomi M Swillens S Gales C. Glycoprotein hormone receptors: link between receptor homodimerization and negative cooperativity. EMBO J (2005) 24(11):1954–64. doi: 10.1038/SJ.EMBOJ.7600686 PMC1142614 15889138

[22] Ulloa-Aguirre A Crépieux P Poupon A Maurel MC Reiter E. Novel pathways in gonadotropin receptor signaling and biased agonism. Rev Endocrine Metab Disord (2011) 12(4):259–74. doi: 10.1007/S11154-011-9176-2 21526415

[23] Jonas KC Chen S Virta M Mora J Franks S Huhtaniemi I. Temporal reprogramming of calcium signalling via crosstalk of gonadotrophin receptors that associate as functionally asymmetric heteromers. Sci Rep (2018) 8(1):2239. doi: 10.1038/s41598-018-20722-5 29396488 PMC5797151

[24] Bakhtyukov AA Derkach KV Gureev MA Dar’in DV Sorokoumov VN Romanova IV. Comparative study of the steroidogenic effects of human chorionic gonadotropin and Thieno[2,3-D]pyrimidine-Based allosteric agonist of luteinizing hormone receptor in young adult, aging and diabetic Male rats. Int J Mol Sci (2020) 21(20):7493. doi: 10.3390/ijms21207493 33050653 PMC7590010

[25] van Straten NCR Schoonus-Gerritsma GG van Someren RG Draaijer J Adang AEP Timmers CM. The first orally active low molecular weight agonists for the LH receptor: thienopyr(im)idines with therapeutic potential for ovulation induction. Chembiochem : A Eur J Chem Biol (2002) 3(10):1023–6. doi: 10.1002/1439-7633(20021004)3:10<1023::AID-CBIC1023>3.0.CO;2-9 12362369

[26] Janovick JA Maya-Núñez G Ulloa-Aguirre A Huhtaniemi IT Dias JA Verbost P. Increased plasma membrane expression of human follicle-stimulating hormone receptor by a small molecule thienopyr(im)idine. Mol Cell Endocrinol (2009) 298(1–2):84–8. doi: 10.1016/j.mce.2008.09.015 PMC2630403 18848862

[27] Heitman LH Oosterom J Bonger KM Timmers CM Wiegerinck PHG IJzerman AP. [3H]Org 43553, the first low-molecular-weight agonistic and allosteric radioligand for the human luteinizing hormone receptor. Mol Pharmacol (2008) 73(2):518–24. doi: 10.1124/MOL.107.039875 17989351

[28] Van Koppen CJ Zaman GJR Timmers CM Kelder J Mosselman S Van De Lagemaat R. A signaling-selective, nanomolar potent allosteric low molecular weight agonist for the human luteinizing hormone receptor. Naunyn-Schmiedeberg’s Arch Pharmacol (2008) 378(5):503–14. doi: 10.1007/s00210-008-0318-3 18551279

[29] Van De Lagemaat R Timmers CM Kelder J Van Koppen C Mosselman S Hanssen RGJM. Induction of ovulation by a potent, orally active, low molecular weight agonist (Org 43553) of the luteinizing hormone receptor. Hum Reprod (Oxford England) (2009) 24(3):640–8. doi: 10.1093/HUMREP/DEN412 19088107

[30] Heitman LH Narlawar R de Vries H Willemsen MN Wolfram D Brussee J. Substituted terphenyl compounds as the first class of low molecular weight allosteric inhibitors of the luteinizing hormone receptor. J Medicinal Chem (2009) 52(7):2036–42. doi: 10.1021/jm801561h 19296599

[31] Heitman LH Kleinau G Brussee J Krause G Ijzerman AP. Determination of different putative allosteric binding pockets at the lutropin receptor by using diverse drug-like low molecular weight ligands. Mol Cell Endocrinol (2012) 351(2):326–36. doi: 10.1016/j.mce.2012.01.010 22269095

[32] Wortmann L Lindenthal B Muhn P Walter A Nubbemeyer R Heldmann D. Discovery of BAY-298 and BAY-899: tetrahydro-1,6-naphthyridine-Based, potent, and selective antagonists of the luteinizing hormone receptor which reduce sex hormone levels *in vivo* . J Medicinal Chem (2019) 62(22):10321–41. doi: 10.1021/acs.jmedchem.9b01382 31670515

[33] Dias JA Bonnet B Weaver BA Watts J Kluetzman K Thomas RM. A negative allosteric modulator demonstrates biased antagonism of the follicle stimulating hormone receptor. Mol Cell Endocrinol (2011) 333(2):143–50. doi: 10.1016/J.MCE.2010.12.023 PMC4491433 21184806

[34] Dias JA Campo B Weaver BA Watts J Kluetzman K Thomas RM. Inhibition of follicle-stimulating hormone-induced preovulatory follicles in rats treated with a nonsteroidal negative allosteric modulator of follicle-stimulating hormone receptor. Biol Reprod (2014) 90(1):19. doi: 10.1095/BIOLREPROD.113.109397 24285717 PMC4435417

[35] Ayoub MA Yvinec R Jégot G Dias JA Poli SM Poupon A. Profiling of FSHR negative allosteric modulators on LH/CGR reveals biased antagonism with implications in steroidogenesis. Mol Cell Endocrinol (2016) 436:10–22. doi: 10.1016/J.MCE.2016.07.013 27424143

[36] Munier M Grouleff J Gourdin L Fauchard M Chantreau V Henrion D. *In vitro* effects of the endocrine disruptor p,p’-DDT on human follitropin receptor. Environ Health Perspect (2016) 124(7):991–9. doi: 10.1289/EHP.1510006 PMC4937862 26895433

[37] Munier M Ayoub M Suteau V Gourdin L Henrion D Reiter E. *In vitro* effects of the endocrine disruptor p,p’DDT on human choriogonadotropin/luteinizing hormone receptor signalling. Arch Toxicol (2021) 95(5):1671–81. doi: 10.1007/S00204-021-03007-1 33638691

[38] Yanofsky SD Shen ES Holden F Whitehorn E Aguilar B Tate E. Allosteric activation of the follicle-stimulating hormone (FSH) receptor by selective, nonpeptide agonists. J Biol Chem (2006) 281(19):13226–33. doi: 10.1074/JBC.M600601200 16540466

[39] Arey BJ Yanofsky SD Claudia Pérez M Holmes CP Wrobel J Gopalsamy A. Differing pharmacological activities of thiazolidinone analogs at the FSH receptor. Biochem Biophys Res Commun (2008) 368(3):723–8. doi: 10.1016/J.BBRC.2008.01.119 18252197

[40] van Koppen CJ Verbost PM van de Lagemaat R Karstens W-JF Loozen HJJ van Achterberg TAE. Signaling of an allosteric, nanomolar potent, low molecular weight agonist for the follicle-stimulating hormone receptor. Biochem Pharmacol (2013) 85(8):1162–70. doi: 10.1016/j.bcp.2013.02.001 23415902

[41] Gerrits MGF Kramer H Galta R Van Beerendonk G Hanssen R Abd-Elaziz K. Oral follicle-stimulating hormone agonist tested in healthy young women of reproductive age failed to demonstrate effect on follicular development but affected thyroid function. Fertility Sterility (2016) 105(4):1056–1062.e4. doi: 10.1016/J.FERTNSTERT.2015.12.017 26769303

[42] Nataraja S Yu H Guner J Palmer S. Discovery and preclinical development of orally active small molecules that exhibit highly selective follicle stimulating hormone receptor agonism. Front Pharmacol (2020) 11:602593. doi: 10.3389/fphar.2020.602593 33519465 PMC7845544

[43] Prabhudesai KS Raje S Dhamanaskar A Modi D Dighe V Contini A. Identification and *in vivo* validation of a 9-mer peptide derived from FSHβ with FSHR antagonist activity. Peptides (2020) 132:170367. doi: 10.1016/J.PEPTIDES.2020.170367 32645381

[44] Smith JS Lefkowitz RJ Rajagopal S. Biased signalling: from simple switches to allosteric microprocessors. Nat Rev Drug Discovery (2018) 17(4):243–60. doi: 10.1038/nrd.2017.229 PMC5936084 29302067

[45] Cao A-M Quast RB Fatemi F Rondard P Pin J-P Margeat E. Allosteric modulators enhance agonist efficacy by increasing the residence time of a GPCR in the active state. Nat Commun (2021) 12(1):5426. doi: 10.1038/s41467-021-25620-5 34521824 PMC8440590

[46] Dror RO Green HF Valant C Borhani DW Valcourt JR Pan AC. Structural basis for modulation of a G-protein-coupled receptor by allosteric drugs. Nature (2013) 503(7475):295–9. doi: 10.1038/nature12595 24121438

[47] Burford NT Watson J Bertekap R Alt A. Strategies for the identification of allosteric modulators of G-protein-coupled receptors. Biochem Pharmacol (2011) 81(6):691–702. doi: 10.1016/J.BCP.2010.12.012 21184747

[48] Ortiz Zacarías NV Lenselink EB IJzerman AP Handel TM Heitman LH. Intracellular receptor modulation: novel approach to target GPCRs. Trends Pharmacol Sci (2018) 39(6):547–59. doi: 10.1016/J.TIPS.2018.03.002 PMC7048003 29653834

[49] Slosky LM Caron MG Barak LS. Biased allosteric modulators: new frontiers in GPCR drug discovery. Trends Pharmacol Sci (2021) 42(4):283–99. doi: 10.1016/J.TIPS.2020.12.005 PMC9797227 33581873

[50] Jäschke H Neumann S Moore S Thomas CJ Colson A-O Costanzi S. A low molecular weight agonist signals by binding to the transmembrane domain of thyroid-stimulating hormone receptor (TSHR) and luteinizing hormone/chorionic gonadotropin receptor (LHCGR). J Biol Chem (2006) 281(15):9841–4. doi: 10.1074/jbc.C600014200 16488885

[51] Newton CL Anderson RC. Pharmacoperones for misfolded gonadotropin receptors. *Handbook of experimental pharmacology* . 245 (2018), 111–34. doi: 10.1007/164_2017_64 29043503

[52] Patil M. Gonadotrophins: the future. J Hum Reprod Sci (2014) 7(4):236–48. doi: 10.4103/0974-1208.147490 PMC4296397 25624659

[53] Nataraja SG Yu HN Palmer SS. Discovery and development of small molecule allosteric modulators of glycoprotein hormone receptors. Front Endocrinol (2015) 6:142. doi: 10.3389/FENDO.2015.00142 PMC4568768 26441832

[54] Kashyap S Parker K Cedars MI Rosenwaks Z. Ovarian hyperstimulation syndrome prevention strategies: reducing the human chorionic gonadotropin trigger dose. Semin Reprod Med (2010) 28(6):475–85. doi: 10.1055/s-0030-1265674 21082506

[55] Tukun FL Olberg DE Riss PJ Haraldsen I Kaass A Klaveness J. Recent development of non-peptide GnRH antagonists. Molecules (Basel Switzerland) (2017) 22(12):2188. doi: 10.3390/MOLECULES22122188 29232843 PMC6149776

[56] Anderson RC Newton CL Millar RP. Small molecule follicle-stimulating hormone receptor agonists and antagonists. Front Endocrinol (2019) 9:757(JAN). doi: 10.3389/FENDO.2018.00757 PMC6352558 30728807

[57] Nataraja S Sriraman V Palmer S. Allosteric regulation of the follicle-stimulating hormone receptor. Endocrinology (2018) 159(7):2704–16. doi: 10.1210/EN.2018-00317 29800292

[58] Aathi MS Kumar C Prabhudesai KS Shanmugarajan D Idicula-Thomas S. Mapping of FSHR agonists and antagonists binding sites to identify potential peptidomimetic modulators. *Biochimica et biophysica acta* . Biomembranes (2022) 1864(4):183842. doi: 10.1016/J.BBAMEM.2021.183842 34954201

[59] Wrobel J Jetter J Kao W Rogers J Di L Chi J. 5-alkylated thiazolidinones as follicle-stimulating hormone (FSH) receptor agonists. Bioorganic Medicinal Chem (2006) 14(16):5729–41. doi: 10.1016/J.BMC.2006.04.012 16675221

[60] Sriraman V Denis D De Matos D Yu H Palmer S Nataraja S. Investigation of a thiazolidinone derivative as an allosteric modulator of follicle stimulating hormone receptor: evidence for its ability to support follicular development and ovulation. Biochem Pharmacol (2014) 89(2):266–75. doi: 10.1016/J.BCP.2014.02.023 24630928

[61] De Pascali F Ayoub MA Benevelli R Sposini S Lehoux J Gallay N. Pharmacological characterization of low molecular weight biased agonists at the follicle stimulating hormone receptor. Int J Mol Sci (2021) 22(18):9850. doi: 10.3390/ijms22189850 34576014 PMC8469697

[62] Sposini S De Pascali F Richardson R Sayers NS Perrais D Yu HN. Pharmacological programming of endosomal signaling activated by small molecule ligands of the follicle stimulating hormone receptor. Front Pharmacol (2020) 11:593492. doi: 10.3389/FPHAR.2020.593492 33329002 PMC7734412

[63] Ji Y Liu P Yuen T Haider S He J Romero R. Epitope-specific monoclonal antibodies to FSHβ increase bone mass. Proc Natl Acad Sci United States America (2018) 115(9):2192–7. doi: 10.1073/PNAS.1718144115 PMC5834707 29440419

[64] Abdennebi L Couture L Grebert D Pajot E Salesse R Remy JJ. Generating FSH antagonists and agonists through immunization against FSH receptor n-terminal decapeptides. J Mol Endocrinol (1999) 22(2):151–9. doi: 10.1677/JME.0.0220151 10194518

[65] Casarini L Simoni M. Recent advances in understanding gonadotropin signaling. Faculty Rev (2021) 10:41. doi: 10.12703/R/10-41 PMC8130412 34046645

[66] Agrawal G Dighe RR. Critical involvement of the hinge region of the follicle-stimulating hormone receptor in the activation of the receptor. J Biol Chem (2009) 284(5):2636–47. doi: 10.1074/jbc.M808199200 19029293

[67] Majumdar R Railkar R Dighe RR. Insights into differential modulation of receptor function by hinge region using novel agonistic lutropin receptor and inverse agonistic thyrotropin receptor antibodies. FEBS Lett (2012) 586(6):810–7. doi: 10.1016/J.FEBSLET.2012.01.052 22309849

[68] Dhar N Mohan A Thakur C Chandra NR Dighe RR. Dissecting the structural and functional features of the luteinizing hormone receptor using receptor specific single chain fragment variables. Mol Cell Endocrinol (2016) 427:1–12. doi: 10.1016/J.MCE.2016.02.022 26940038

[69] Crepin R Veggiani G Djender S Beugnet A Planeix F Pichon C. Whole-cell biopanning with a synthetic phage display library of nanobodies enabled the recovery of follicle-stimulating hormone receptor inhibitors. Biochem Biophys Res Commun (2017) 493(4):1567–72. doi: 10.1016/J.BBRC.2017.10.036 29017919

[70] Van Straten NCR Van Berkel THJ Demont DR Karstens WJF Merkx R Oosterom J. Identification of substituted 6-amino-4-phenyltetrahydroquinoline derivatives: potent antagonists for the follicle-stimulating hormone receptor. J Medicinal Chem (2005) 48(6):1697–700. doi: 10.1021/JM049676L 15771412

[71] Jiang X Fischer D Chen X McKenna SD Liu H Sriraman V. Evidence for follicle-stimulating hormone receptor as a functional trimer. J Biol Chem (2014) 289(20):14273–82. doi: 10.1074/JBC.M114.549592 PMC4022893 24692546

[72] Waghu FH Desai K Srinivasan S Prabhudesai KS Dighe V Venkatesh KV. FSHR antagonists can trigger a PCOS-like state. Syst Biol Reprod Med (2022) 68(2):129–37. doi: 10.1080/19396368.2021.2010837 34967272

[73] Gomes I Jordan BA Gupta A Rios C Trapaidze N Devi LA. G Protein coupled receptor dimerization: implications in modulating receptor function. J Mol Med (Berlin Germany) (2001) 79(5–6):226–42. doi: 10.1007/S001090100219 11485015

[74] Gomes I Ayoub MA Fujita W Jaeger WC Pfleger KDG Devi LA. G Protein-coupled receptor heteromers. *Annual review of pharmacology and toxicology* . 56 (2016), 403–25. doi: 10.1146/ANNUREV-PHARMTOX-011613-135952 PMC5147582 26514203

[75] Agwuegbo UC Jonas KC. Molecular and functional insights into gonadotropin hormone receptor dimerization and oligomerization. Minerva Ginecologica (2018) 70(5):539–48. doi: 10.23736/S0026-4784.18.04287-9 PMC7086338 30226027

[76] Casarini L Santi D Simoni M Potì F. Spare’ luteinizing hormone receptors: facts and fiction. Trends Endocrinol Metab (2018) 29,4:208–17. doi: 10.1016/j.tem.2018.01.007 29429918

[77] Birdsall NJM. Class a GPCR heterodimers: evidence from binding studies. Trends Pharmacol Sci (2010) 31(11):499–508. doi: 10.1016/J.TIPS.2010.08.003 20870299

[78] Guan R Wu X Feng X Zhang M Hébert TE Segaloff DL. Structural determinants underlying constitutive dimerization of unoccupied human follitropin receptors. Cell Signalling (2010) 22(2):247–56. doi: 10.1016/J.CELLSIG.2009.09.023 PMC2787667 19800402

[79] Jonas KC Fanelli F Huhtaniemi IT Hanyaloglu AC. Single molecule analysis of functionally asymmetric G protein-coupled receptor (GPCR) oligomers reveals diverse spatial and structural assemblies. J Biol Chem (2015) 290(7):3875–92. doi: 10.1074/jbc.M114.622498 PMC4326798 25516594

[80] Navarro G Ferré S Cordomi A Moreno E Mallol J Casadó V. Interactions between intracellular domains as key determinants of the quaternary structure and function of receptor heteromers. J Biol Chem (2010) 285(35):27346–59. doi: 10.1074/jbc.M110.115634 PMC2930733 20562103

[81] Ferré S Casadó V Devi LA Filizola M Jockers R Lohse MJ. G Protein-coupled receptor oligomerization revisited: functional and pharmacological perspectives. Pharmacol Rev (2014) 66(2):413–34. doi: 10.1124/PR.113.008052 PMC3973609 24515647

[82] Amsterdam A Berkowitz A Nimrod A Kohen F. Aggregation of luteinizing hormone receptors in granulosa cells: a possible mechanism of desensitization to the hormone. Proc Natl Acad Sci United States America (1980) 77(6):3440–4. doi: 10.1073/PNAS.77.6.3440 PMC349632 6251459

[83] Johnson GP Jonas KC. Mechanistic insight into how gonadotropin hormone receptor complexes direct signaling†. Biol Reprod (2020) 102(4):773–83. doi: 10.1093/biolre/ioz228 PMC7608586 31882999

[84] Casadó V Cortés A Mallol J Pérez-Capote K Ferré S Lluis C. GPCR homomers and heteromers: a better choice as targets for drug development than GPCR monomers? Pharmacol Ther (2009) 124(2):248–57. doi: 10.1016/J.PHARMTHERA.2009.07.005 PMC9386294 19664655

[85] Calebiro D Rieken F Wagner J Sungkaworn T Zabel U Borzi A. Single-molecule analysis of fluorescently labeled G-protein-coupled receptors reveals complexes with distinct dynamics and organization. Proc Natl Acad Sci United States America (2013) 110(2):743–8. doi: 10.1073/PNAS.1205798110 PMC3545784 23267088

[86] Rivero-Müller A Chou YY Ji I Lajic S Hanyaloglu AC Jonas K. Rescue of defective G protein-coupled receptor function *in vivo* by intermolecular cooperation. Proc Natl Acad Sci United States America (2010) 107(5):2319–24. doi: 10.1073/PNAS.0906695106 PMC2836644 20080658

[87] Mazurkiewicz JE Herrick-Davis K Barroso M Ulloa-Aguirre A Lindau-Shepard B Thomas RM. Single-molecule analyses of fully functional fluorescent protein-tagged follitropin receptor reveal homodimerization and specific heterodimerization with lutropin receptor. Biol Reprod (2015) 92(4):100. doi: 10.1095/BIOLREPROD.114.125781 25761594 PMC4643952

[88] Casarini L Reiter E Simoni M. β-arrestins regulate gonadotropin receptor-mediated cell proliferation and apoptosis by controlling different FSHR or LHCGR intracellular signaling in the hGL5 cell line. Mol Cell Endocrinol (2016) 437:11–21. doi: 10.1016/j.mce.2016.08.005 27502035

[89] Casarini L Lazzaretti C Paradiso E Limoncella S Riccetti L Sperduti S. Membrane estrogen receptor (GPER) and follicle-stimulating hormone receptor (FSHR) heteromeric complexes promote human ovarian follicle survival. IScience (2020) 23(12):101812. doi: 10.1016/j.isci.2020.101812 33299978 PMC7702187

[90] Itskovitz J Boldes R Levron J Erlik Y Kahana L Brandes JM. Induction of preovulatory luteinizing hormone surge and prevention of ovarian hyperstimulation syndrome by gonadotropin-releasing hormone agonist. Fertility Sterility (1991) 56(2):213–20. doi: 10.1016/S0015-0282(16)54474-4 1906406

[91] van de Lagemaat R Raafs BC van Koppen C Timmers CM Mulders SM Hanssen RGJM. Prevention of the onset of ovarian hyperstimulation syndrome (OHSS) in the rat after ovulation induction with a low molecular weight agonist of the LH receptor compared with hCG and rec-LH. Endocrinology (2011) 152(11):4350–7. doi: 10.1210/en.2011-1077 21896671

[92] Goodarzi MO Dumesic DA Chazenbalk G Azziz R. Polycystic ovary syndrome: etiology, pathogenesis and diagnosis. Nat Rev Endocrinol (2011) 7(4):219–31. doi: 10.1038/nrendo.2010.217 21263450

[93] Brynhildsen J. Combined hormonal contraceptives: prescribing patterns, compliance, and benefits versus risks. Ther Adv Drug Saf (2014) 5(5):201–13. doi: 10.1177/2042098614548857 PMC4212440 25360241

[94] Casarini L Crépieux P Reiter E Lazzaretti C Paradiso E Rochira V. FSH for the treatment of Male infertility. Int J Mol Sci (2020) 21(7):2270. doi: 10.3390/IJMS21072270 32218314 PMC7177393

